# Tularemia: Historical Perspectives and Current Challenges of a Re-Emerging Zoonosis

**DOI:** 10.3390/biomedicines14030695

**Published:** 2026-03-17

**Authors:** Maria Di Spirito, Chiara Pascolini, Simonetta Salemi, Ferdinando Spagnolo, Vincenzo Luca, Filippo Molinari, Orr Rozov, Florigio Lista, Raffaele D’Amelio, Silvia Fillo

**Affiliations:** 1 Defense Institute for Biomedical Sciences, 00184 Rome, Italy; maria.dispirito@persociv.difesa.it (M.D.S.); chiara.pascolini@persociv.difesa.it (C.P.); cadip.medsitaw@isbd.difesa.it (F.S.); vincenzoluca85@gmail.com (V.L.); florigio.lista@esercito.difesa.it (F.L.); 2 Dipartimento di Sanità Pubblica e Malattie Infettive, Sapienza Università di Roma, 00185 Rome, Italy; 3 Dipartimento di Scienze Cliniche e Medicina Traslazionale, Università di Roma Tor Vergata, 00133 Rome, Italy; 4 Azienda Ospedaliero-Universitaria Sant’ Andrea, 00189 Roma, Italy; simonetta.salemi@ospedalesantandrea.it; 5 Dipartimento di Chimica, Biologia e Biotecnologie, Università di Perugia, 06123 Perugia, Italy; 6 Independent Researcher, 00162 Rome, Italy; raffaele.damelio@gmail.com

**Keywords:** tularemia, *Francisella tularensis*, zoonosis, vector, One Health, vaccines, protective antibodies, antibiotics

## Abstract

Tularemia is a plague-like, potentially fatal zoonosis caused by the coccobacillus *Francisella tularensis*. It was discovered at the beginning of the last century in the United States and was soon recognized in Japan and in the former Soviet Union as the cause of clinical conditions that had been known for one and two centuries, respectively. More than 250 animal species are susceptible to infection, with rodents and lagomorphs serving as key reservoirs, and several vectors may transmit the disease, mainly ticks and mosquitoes. Humans are incidental hosts and are infected primarily by two *F. tularensis* subspecies, *tularensis* and *holarctica*: the former is more severe and is found almost exclusively in North America, whereas the latter is distributed throughout the Northern Hemisphere, mainly in Europe and Asia. Tularemia is highly infectious; therefore, diagnostic cultures should be handled in biosafety level 3 laboratories. Nevertheless, interhuman transmission is exceedingly rare. Although tularemia is relatively uncommon, it shows a re-emerging pattern at the global level, particularly in Europe. As with plague, mitigation may be more effectively achieved through a One Health approach. Neither approved vaccines nor therapeutic antibodies are currently available, whereas aminoglycoside, tetracycline, and quinolone antibiotics are effective. Owing to its high infectivity, its ease of transmission by inhalation, its clinical severity, with a prolonged and debilitating course, and its potential lethality, *F. tularensis* has long been considered a potential biological weapon, particularly if antibiotic-resistant strains were used. Although natural antibiotic resistance has not been described to date, research programs aimed at obtaining resistant strains have been conducted. It has been suggested that the disease was already present in the Middle East during the second millennium BC; should this hypothesis be confirmed by paleogenomic studies, plague and tularemia would have coexisted for more than three millennia, with plague masking the less severe tularemia. Many challenges related to tularemia are still unresolved.

## 1. Introduction

Tularemia is a potentially fatal, plague-like zoonotic disease caused by the coccobacillus *F. tularensis*, which may be transmitted to humans by a wide range of susceptible animals, particularly rodents and lagomorphs, and/or by arthropod vectors [1]. The disease is rare; however, in recent years it has shown a re-emerging epidemiological pattern [2], particularly in Europe [1]. Although human-to-human transmission has been observed only very rarely [3], *F. tularensis* is among the most infectious bacterial agents, with as few as 10 microorganisms being sufficient to cause disease in humans. Because of this characteristic and the severity of the associated clinical manifestations, *F. tularensis* has long been considered a potential biological weapon [4].

## 2. History

The documented history of tularemia is comparatively recent and, in its early phase, unfolded entirely in the United States [5]. The disease was first described in the early twentieth century by George Walter McCoy, then Director of the U.S. Public Health Service Plague Laboratory. During surveillance activities in California, he investigated a “plague-like” illness affecting ground squirrels and identified microorganisms that did not correspond to *Yersinia pestis* [6]. Subsequent studies allowed more precise characterization of the agent. The newly identified bacterium was designated *Bacterium tularense,* a name derived from Tulare County, California, where it had first been detected [7].

The first human case of tularemia was described in 1914 in a man presenting with conjunctivitis and lymphadenopathy [8]. In 1919, Edward Francis moved from Washington, DC, to Utah as a public health officer to study the so-called “deer-fly fever,” in which he identified *Bacterium tularense* as the etiological agent, thereby initiating extensive and systematic investigations into the disease in rodents, lagomorphs, and humans. He also coined the term “tularemia”, noting that *B. tularense* could be isolated from blood, and proposed culture modifications to optimize the growth of the microorganism. In recognition of his contributions, the scientific community accepted the proposal by Dorofeev [9,10] to rename the organism *F. tularensis* [11].

Francis and all five of his collaborators engaged in laboratory work and/or autopsies contracted tularemia, highlighting the very high infectivity of the microorganism, despite the fact that interhuman transmission is exceedingly rare [12]. In 1925, Edward Francis collaborated with Hachiro Ohara, who had described a tularemia-like syndrome in Japan that was subsequently confirmed to be tularemia [13]. In the former Soviet Union, tularemia was first identified in 1929, when Zarkhi, a clinical researcher who fell ill after necropsying guinea pigs inoculated with material from the buboes of patients affected by an unexplained disease, sent his serum to George McCoy, who diagnosed tularemia. Russian researchers later retrospectively reinterpreted cases of the so-called “Siberian ulcer” reported in the eighteenth and nineteenth centuries as tularemia rather than cutaneous anthrax, based on the observed low mortality rate (approximately 2%) [14].

## 3. *Francisella tularensis*

*F. tularensis* is a rod-shaped or coccobacillary, non-motile, non-spore-forming, aerobic, facultative intracellular [15] Gram-negative bacterium that requires cysteine or cystine for growth [11]. It can be cultivated on cystine heart blood agar supplemented with 1% hemoglobin, where convex, opalescent colonies appear after 2–4 days of incubation [11]. *F. tularensis* produces acid but no gas from a limited range of carbohydrates [11], whereas *F. novicida* does not [16].

The taxonomic history of *F. tularensis* is complex. Initially, it was included in the genus *Bacterium*. Subsequently, it was included in the genus *Pasteurella*, and then provisionally in the genus *Brucella*. Only in 1947, more than 35 years after its discovery, was it proposed, on the basis of biochemical tests, that *F. tularensis* should constitute the sole member of a new genus, *Francisella* [9,16]. Subsequently, analyses of fatty acid composition and 16S rDNA sequences led to the recognition of two species within the genus *Francisella*: *F. tularensis* and *F. philomiragia* [4].

Currently, the genus *Francisella* is considered to include several species and subspecies. *F. tularensis* comprises three recognized subspecies, *tularensis* (type A), *holarctica* (type B), and *mediasiatica*, and, according to some authors, possibly a fourth, *novicida*. Although *F. novicida* shares ≥97.7% genomic identity with *F. tularensis* subsp. *tularensis*, *holarctica*, and *mediasiatica*, substantial functional and structural differences exist; consequently, there is no consensus on whether *F. novicida* should be classified as a subspecies of *F. tularensis* or as an independent species [17,18]. Human tularemia cases are almost entirely attributable to the subspecies *tularensis* and *holarctica*: the former is more virulent and occurs almost exclusively in North America, whereas the latter is less severe and is distributed throughout the Northern Hemisphere [11].

Based on multilocus variable-number tandem repeat analysis (MLVA), *F. tularensis* subsp. *tularensis* (type A) can be divided into two lineages, A.I and A.II, with distinct geographic distributions: A.I is found predominantly in the central and eastern United States, whereas A.II is present west of the 100th meridian [14,19]. Lineage A.I has been further subdivided into A.Ia and A.Ib, the latter being associated with more severe disease than A.Ia, A.II, and type B strains [20]. Whole-genome sequencing (WGS) and canonical single-nucleotide polymorphism (canSNP) genotyping have identified four major lineages within *F. tularensis* subsp. *holarctica* [14,18]: clades B.4 and B.6, which include erythromycin-sensitive biovar I (EryS); clade B.12, which contains erythromycin-resistant biovar II (EryR); and the biovar *japonica*, characterized by its ability to ferment glycerol and assigned to clade B.16 [21,22].

*F. tularensis* subsp. *mediasiatica* is present in Central Asia and has not been reported to cause human infection [23]. *F. novicida*, which is commonly found in aquatic environments, is highly pathogenic for animals but has been only rarely reported in humans, mainly in immunocompromised or elderly individuals; in addition to North America, it has also been isolated in Australia [4,24]. Other species, including *F. philomiragia*, *F. hispaniensis*, *F. opportunistica*, as well as *F. novicida*, may occasionally infect humans. *F. salimarina* and *F. halioticida* have been reported to infect humans only very rarely, whereas other species, such as *F. persica*, *F. noatunensis*, *F. endocilophora*, and *F. uliginis*, have not been described as human pathogens [23].

*F. tularensis* has a characteristic composition of long-chain saturated and unsaturated fatty acids that can be identified by gas–liquid chromatography, a method that may also be used for diagnostic purposes [11]. Diagnosis can be performed by culture; however, this approach is hazardous and should be carried out only in biosafety level 3 laboratories, given that *F. tularensis* is one of the most transmissible infectious agents. Consequently, molecular techniques, such as polymerase chain reaction (PCR) [25], and immunological tests [26] are more commonly used for diagnosis.

## 4. Epidemiology

The epidemiology of tularemia shows an irregular yet persistent distribution pattern, with major outbreaks reported mainly in temperate areas of the Northern Hemisphere. Since the early twentieth century, cases have been documented in North America, Europe, and Asia. Over time, the disease has extended to additional regions of the Northern Hemisphere and, more rarely, to parts of the Southern Hemisphere. Reports from Australia [24,27] and Tasmania [28], limited to *F. novicida*, suggest a gradual geographic expansion [2]. Following its initial identification, tularemia spread widely, reaching a peak during the Second World War. The most extensive burden was recorded in the former Soviet Union, where approximately 100,000 cases per year were reported in the 1940s [29]. Armed conflict appears to have amplified transmission. The disruption of agricultural activities, deteriorating sanitation, and consequent surges in rodent populations created ecological conditions favorable to large-scale outbreaks [29]. In the decades after the war, the global incidence declined steadily throughout the second half of the twentieth century. More recently, however, several countries have documented renewed activity. In Europe in particular, outbreaks have occurred in areas not previously regarded as endemic, including Spain in 1997 and Kosovo in 1999–2000, with the latter taking place in the context of local armed conflict.

The subspecies involved in human infection include *F. tularensis* subsp. *tularensis* (type A), which tends to cause more severe clinical manifestations and predominates in North America, and *F. tularensis* subsp. *holarctica* (type B), which is mainly distributed in Eurasia [30]. Tularemia exhibits marked seasonality, with most cases occurring between July and November. This pattern corresponds to increased activity of vectors such as ticks and mosquitoes, which elevates the risk of human exposure. Despite annual fluctuations, infections are concentrated in summer and autumn, when human contact with vector-infested environments is more frequent [31]. In the United States, incidence shows a bimodal distribution, with a first peak in summer associated with tick bites and a second peak between November and February linked to hunting activities [32].

In Northern Europe, countries such as Sweden and Finland report hundreds of human cases annually, with seasonal peaks in summer and autumn, likely related to outdoor activities and increased interactions between humans, animals, and vectors [31]. Surveillance data indicate rising trends. In Denmark, the incidence increased from 0–4 to 14–25 cases per year in the last five years (with 25 cases in 2023) [31], while in Germany, 152 human cases were reported between 2012 and 2022 (including five fatalities) in the Baden-Wuerttemberg state, corresponding to an average annual increase of approximately 20% [33,34]. A Europe-wide analysis from 1992 to 2012 documented 18,343 cases, with Sweden accounting for 25% and Finland for 22% of the total, and showing recurrent peaks in 2000, 2003, and 2010 [35]. Clinical presentations include ulceroglandular, glandular, pneumonic, typhoidal, and oropharyngeal forms, with generally low, but non-negligible, mortality in some populations.

Recent European data reveal a clear trend of reemergence. In 2019, 1463 confirmed cases were reported across 21 EU/EEA countries, with an overall incidence of approximately 0.3 cases per 100,000 inhabitants and more than half of cases occurring in Sweden [36]. In 2021, 876 cases were confirmed, representing a 33% increase compared with 2020 [37]. According to EU One Health data for 2023, 1185 confirmed cases were reported, corresponding to an incidence of 0.27 per 100,000 inhabitants, an 89.3% increase from 0.14 in 2022, with most cases concentrated in Sweden, Finland, Austria, and Hungary [38]. In the west of France, the cases of tularemia doubled in 2018 compared with the previous year with many pneumonic forms, thus causing suspicion of a substantial environmental tularemia contamination. An analysis of the water in that area carried out in July 2019 and January 2020 allowed the observation of the presence of *F. novicida*, which is a well-known contaminant of water, particularly brackish water, but also of *F. holarctica*, whose presence in the aquatic environment contributes to the endemicity of that area [39]. In Switzerland, 61 cases of tularemia were observed in the period 2004–2013 versus 430 cases in the period 2014–2022, a 7-fold increase; among susceptible animals 3 cases were observed in 2011 versus 10 in 2021, a 3.3-fold increase. The causes of this recent increment are unknown; however, the authors of the report strongly recommend following a One Health strategy in order to provide a possible explanation [40]. The analysis of the European Centre for Disease Prevention and Control (ECDC) shows that in the period 2019–2023 [38], Austria, France, and Germany experienced a 3.75-fold, 3.05-fold, and 1.652-fold increase in tularemia cases, respectively, compared to the period 2015–2019 [36]. Such a trend of reemerging infectious disease in Northern–Central Europe is accompanied by a tularemia spread from the eastern to the western part of the continent, even long-distance, as suggested by genetic studies on human sera collected from different European countries along an east–west geographic gradient and across a 65-year temporal range spanning from 1947 to 2012 [41]. The results show in the eastern countries a greater strain variability, reflecting an older local presence, versus a more monophyletic pattern in western countries, reflecting a more recent colonization [42]. Moreover, this observation implies that, probably in association with climate modifications [43], the epidemiology of tularemia is deeply changing by incorporating new endemic hotspots not previously affected, thus strengthening once more the need for careful integrated surveillance across human, animal, entomological, and environmental domains according to the One Health approach to predict possible outbreaks and implement prophylactic or mitigation measures. The reasons for tularemia re-emergence are still not fully understood, but certainly include an improved surveillance system, considering that tularemia is currently a generally notifiable disease in Europe, even though the rate of underreporting is still quite high [33,44], as frequently happens with passive surveillance of rare diseases that are difficult to diagnose.

This suggestion is further supported by serological studies. A recent systematic review and meta-analysis of human seroprevalence studies conducted between 1951 and 2023 estimated a pooled seroprevalence of 3.7% (95% CI 2.7–5.1) and found that approximately 84% of seropositive individuals had no history of clinically recognized tularemia, indicating that the majority of infections may remain subclinical or undiagnosed and therefore escape routine notification systems [45]. In Germany, two serological studies carried out five years apart, the first one in 2004 on 6632 subjects representative of the whole country and the second in 2009 on 2416 human sera collected in one city of the Baden-Wüerttemberg federal state, provided two very different results, with 0.23% in the first study [46] and 2.3% in the second [43]. Such a 10-fold difference was not due to methodological reasons (data were obtained in the same laboratory with the same techniques), but probably to the relatively high rate in the second study of risk subjects for occupational exposure or exposure to infectious animals. However, the higher-than-expected data in this study, considering that it was carried out in a presumed low-risk area, imply that tularemia may be underestimated in Germany. A serological study carried out in Austria in 2014 on 526 serum samples from military personnel and adult civilians provided 3 positive sera (0.5%) [47]. A very recent seroepidemiological study in Western Austria carried out on 3008 blood donors provided a result of 2% of seropositive individuals with more than 1% of additional borderline subjects against a mean annual incidence of notified cases of 0.3/100,000 in the previous decade in Western Austria, and an estimated incidence of 29/100,000 cases, thus strongly highlighting once more a marked rate of underreporting or underdiagnosis [48]. Notably, in this latter study the incidence derived from seroprevalence was more than thirty-fold higher than the officially notified incidence (0.3/100,000 per year), further supporting the concept that surveillance based exclusively on clinically apparent cases may capture only a small fraction of total infections. Moreover, the net increase of seropositivity in 11 years from 0.5% in a population of the whole Austria [47] to 2% in a population of the Western Austria [48], which is generally considered a low-prevalence area for tularemia, is further evidence of the markedly increased prevalence of *F. tularensis* in Austria. A serological study performed among 2875 subjects from five Northeastern French regions some of which border the German federal state of Baden-Württemberg, where a seroprevalence of 2.32% for tularemia was reported in the city of Leutkirk [43], found 164 positive samples, a rate of 5.7% [49], mainly in forestry workers. These serological studies confirm the spread of tularemia along an east–west gradient, reaching and contaminating areas previously considered free of infection. The relevance of the information provided by the seroepidemiological studies for tularemia of blood donors or subjects from the general population is also witnessed by the results observed in other European countries, where percentages of seropositive subjects ranging from 1.5% in Finland [50], to 3.2% in Poland [51], up to 4% in Slovakia [52], were observed.

Serological and genomic studies in mammals, according to the One Health approach, are of paramount importance, not only to identify the geographic areas and animal hosts colonized by *F. tularensis*, but also to estimate the duration of colonization, based on the high phylogenetic diversity [53], thus providing the scientific tools for reconstructing the temporal kinetics of the east–west tularemia spread. Serological surveillance of captive and wild animals in Germany allowed to establish that in captive animals only 3/1122 (0.3%) were positive, whereas among 1353 wild foxes, raccoon dogs, and wild boars collected during the period 2005–2009 in the Brandenburg federal state 101 (7.5%) were positive. Foxes and raccoon dogs were considered sentinel indicators of tularemia [54].

Over the past two years, further reemergence has been observed. In the United States, 220 cases were reported in 2024, with an additional 15 confirmed cases in early 2025 [2]. In Europe, outbreaks have continued, such as in Austria, where 117 cases and one death were reported during the same period [2]. Overall, tularemia has been reported in 35 countries, with the highest numbers of cases recorded in the United States, Turkey, Sweden, Finland, and Japan [2]. In France, 2024 was the year with the highest number of tularemia case notifications since 2002, 150 cases, with 45% pneumonic clinical forms, and 10 cases in Alsace, 30% of the 33 cases reported in Northeastern France [55].

In the Middle East, recent studies indicate a human seroprevalence of 6.2% in at-risk populations, with evidence of *F. tularensis* detected in environmental samples (~5.8%), vectors such as ticks (~2.5%), and rodents (~2.0%), suggesting persistent but underestimated endemicity [30]. In Scandinavia, increases in human cases have been associated with population surges of lemmings, natural hosts of the bacterium, while in other European regions outbreaks appear to result from a combination of favorable ecological and environmental factors [31].

Taken together, these findings confirm that tularemia is a reemerging zoonosis, with rising trends particularly in Europe underscoring the need for active epidemiological surveillance, integrated One Health approaches, and targeted interventions to prevent outbreaks in vulnerable human and animal populations.

## 5. Ecology and Transmission

*F. tularensis* exhibits a highly complex ecology, sustained by multi-level enzootic networks involving numerous vertebrate hosts, arthropod vectors, and environmental compartments. The bacterium has been isolated from more than 250 animal species [23], making it one of the zoonotic pathogens with one of the broadest known host ranges [1,56].

Rodents and lagomorphs are suspected to be key reservoirs and amplifying hosts. In Europe and Asia, these include voles (*Microtus* spp.), water voles (*Arvicola terrestris*), muskrats (*Ondatra zibethicus*), and beavers (*Castor* spp.), while in North America they include cottontail rabbits (*Sylvilagus* spp.) and hares (*Lepus* spp.) [1,57,58]. Other vertebrates, such as wild carnivores (foxes, mustelids), ungulates, and domestic animals (dogs and cats), can act as incidental hosts or bridges between wildlife and humans [1,59,60,61,62]. In the United States, 3.3% of human cases of tularemia occurring during the period 2006–2016 were attributed to dogs through bites, scratches, licks, transport of dead animals, or introduction of infected ticks into domestic settings, highlighting that the risk extends beyond natural environments into anthropized areas [63]. Moreover, cats seem to contribute to tularemia more frequently than dogs, as observed in the United States: 8 cat-associated human cases out of 106 (7.5%) *F. tularensis* isolates collected during 1998–2012 [64]. Some professions are particularly exposed to the risk of infection with *F. tularensis*, such as veterinarians, hunters, farmers, butchers, and laboratory workers. In a survey carried out during the period 2012–2022 in the US, the veterinarians’ professional risk for tularemia was four-fold higher for cats than for dogs, considering that 16 cases of cat-associated versus 4 dog-associated infections in veterinarians were observed [65].

Although birds show limited clinical susceptibility, they contribute to the geographic spread of the pathogen by transporting infected ticks shared with rodents and lagomorphs [1,66,67]. In particular, birds have been considered potential drivers of long-distance spread of *F. tularensis.*

Transmission cycles of *F. tularensis* differ depending on the ecosystem and the subspecies involved. Subspecies *tularensis* (type A) is typically associated with a terrestrial cycle, in which wild lagomorphs, such as rabbits and hares, act as amplifying hosts and arthropods as dissemination vectors. Type A.I occurs in the Central and Eastern United States, primarily linked to eastern cottontails, whereas type A.II occurs in the Western United States, involving desert cottontails [14]. Subspecies *holarctica* (type B) is mainly associated with an aquatic cycle involving semi-aquatic rodents such as beavers, muskrats, and voles, with contamination of water bodies via urine, feces, and carcasses, and is especially common in Eurasia [14]. Terrestrial rodents, rats, raccoons, squirrels, and wild rabbits also seem to act as natural reservoirs of type B, even though our knowledge of this topic is still largely incomplete and no relationship between a vertebrate species and type B lineages appears to exist [1,14].

Animal-to-animal transmission can occur through skin contact, respiratory routes, or ingestion of contaminated material. Hematophagous arthropods (ticks, flies, mites, midges, fleas, mosquitoes, and lice) play a central role, with ticks of the genera *Dermacentor*, *Ixodes*, *Amblyomma*, and *Ornithodoros* acting as biological vectors, maintaining *F. tularensis* through transstadial and transovarial transmission and sometimes serving as reservoirs themselves [14,16,68,69]. In biological transmission, the pathogen replicates within the vector, which actively transfers it to the host through biting, functioning as both vector and reservoir, analogous to fleas in *Yersinia pestis* transmission [14,16,68]. Mechanical transmission occurs when arthropods such as horseflies (*Tabanidae*) carry the bacterium on body surfaces or mouthparts without replication, enabling short-term infection via bites or contact with contaminated excreta [20]. Ticks play a major role in type A transmission in North America and much of Eurasia but appear to be less important in Scandinavia, where tularemia incidence is higher than in other parts of Europe and mosquitoes are mainly involved in transmission [14]. Mosquitoes can acquire infection at the larval stage in aquatic environments, acting as significant vectors in specific ecological contexts, such as Scandinavia [70,71]. Because tularemia often causes septicemia and infected animals frequently exhibit lethargy, transmission via hematophagous vectors is further facilitated. In addition to vector-borne transmission, infection can occur through ingestion of infected carcasses or contaminated water, or through inhalation of dust, feces, or aerosols containing the bacterium, potentially resulting in pulmonary disease [41].

A central aspect of *F. tularensis* ecology is its ability to persist in the environment. Field and experimental studies have documented its presence in soil as well as in fresh and brackish water [39,62,72,73,74]. In these habitats, the bacterium can persist through association with biofilms and with free-living amoebae, which serve as environmental reservoirs and promote dissemination among aquatic animals [71,75]. Environmental contamination occurs mainly via urine, feces, and carcasses of infected animals. Under experimental conditions, *F. tularensis* can survive for weeks or months in nutrient-free water at temperatures between 4 and 20 °C and at varying salinities, likely due to transition into a viable but non-culturable (VBNC) state, interaction with amoebae, and biofilm formation [39,76,77,78,79,80,81]. It has been estimated that a single infected aquatic rodent can contaminate up to 500,000 L of water, confirming aquatic systems to be potent ecological amplifiers of infection [82]. Further evidence of the high infective potential of *Francisella* in liquid environments was observed in Germany in 2016, when an outbreak of six cases of oropharyngeal tularemia occurred after ingestion of a small amount of grape must (fermented juice) from a 730-L batch contaminated by an estimated 10^9^–10^10^ bacteria originating from the carcass of a rodent. Of the eight individuals who consumed the contaminated must, six developed tularemia, corresponding to an attack rate of 75% [83].

This ecological plasticity allows *F. tularensis* to adapt to terrestrial, aquatic, and mixed transmission cycles, as demonstrated in the former Soviet Union, where six distinct epidemiological scenarios—meadow-field, steppe, forest, floodplain, riverine, and desert—were described, each characterized by specific combinations of hosts, vectors, and human exposure routes [14]. Collectively, these models highlight the pathogen’s extraordinary capacity to persist in diverse ecosystems, simultaneously exploiting biological and mechanical vectors, vertebrate reservoirs, and environmental compartments.

Tularemia represents a paradigmatic environment-dependent zoonosis, with human infection frequently acquired through interaction with contaminated ecosystems, minor skin injuries, recreational activities, or consumption of contaminated water, underscoring the environment as an active epidemiological compartment rather than a passive vehicle [84,85,86]. Direct contact with infected vertebrates, whether alive or dead, represents one of the most relevant transmission mechanisms to humans, particularly in hunting, wildlife handling, and meat preparation contexts. Infection can occur through contact with skin, blood, or tissues, or through consumption of raw or undercooked meat, illustrating how food-related and hunting practices may facilitate pathogen spillover from natural ecosystems to humans [87].

The breadth of involved vectors reflects the ecological versatility of *F. tularensis*. In North America, tick bites are the predominant route of human transmission, whereas in Sweden and Finland outbreaks are mainly mosquito-borne, with infection acquired at the larval stage in aquatic habitats [70,88,89]. Ticks are considered the primary biological vectors in most endemic areas, while horseflies act as mechanical vectors, transmitting the bacterium via contaminated mouthparts without long-term survival [20]. Regional differences, such as the involvement of horseflies in Utah compared with the dominant role of ticks in other US states, highlight the importance of the local ecological context in shaping transmission cycles [1].

Transmission patterns also vary across regions. In the United States, Scandinavia, and Russia, vector-borne transmission predominates, whereas in Western and Central Europe direct contact with infected animals and ingestion of contaminated food or water are more common. In Turkey, transmission is almost exclusively waterborne, with outbreaks linked to consumption of contaminated spring water and a predominance of oropharyngeal forms, underscoring the critical role of water infrastructure and natural resource management in determining human risk [90,91].

Anthropogenic activities amplify natural transmission cycles of *F. tularensis*. Hunting, agricultural expansion, land use changes, wildlife management, and increased meat consumption enhance direct contact with infected animals and vectors. Activities such as lawn mowing, hay stacking, or soil work can aerosolize contaminated biological material, leading to respiratory infections, as demonstrated by outbreaks linked to accidental fragmentation of animal carcasses during agricultural or gardening activities [92,93].

## 6. One Health

The increasing trend in tularemia cases may reflect ongoing transformations in environmental, animal, and human transmission cycles, as well as improved diagnostic sensitivity and reporting. This interconnected system implies that human health cannot be considered separately from animal and environmental health; therefore, a One Health approach is necessary to effectively understand and manage tularemia by integrating the risks emerging from each compartment [33,35,62,63].

The intensification of anthropogenic activities that alter or invade natural environments contributes significantly to the activation and amplification of zoonotic outbreaks. Urban expansion, land use for agricultural or infrastructure purposes, deforestation, hunting, and wildlife harvesting increase contact between humans, wildlife, and arthropod vectors, creating opportunities for pathogen spillover, such as with *F. tularensis* [35,62]. The destruction and invasion of natural habitats force wild animals to search for food and shelter in peri-urban or urban areas, reducing the ecological distance between animals and humans and increasing the risk of spillover [62]. Both occupational and recreational activities have progressively encroached upon natural spaces, significantly increasing infection risk. For instance, hunters, veterinarians, butchers, and agricultural workers, who are often in close contact with animals and vectors such as ticks, are at heightened risk of tularemia infection, as observed in several European countries [94]. Hunters are exposed to *F. tularensis* through direct contact with infected animals, consumption of wild game, and exposure to vectors such as ticks. In Germany, 1.7% of 286 hunters tested positive for *F. tularensis*, compared with only 0.2% of the general population (OR ≈7.7, *p* < 0.001). In Southeastern Austria, 5 of 149 hunters (3.35%) tested positive, whereas none of the 50 urban residents did. In Québec, 2.4% of trappers tested positive, compared with 0.6% of controls [62,95,96]. High hare densities in certain areas of Europe, such as the Czech Republic, France, and Germany, have been linked to peaks of infection among hunters, highlighting how wildlife habitat encroachment and increased contact with local fauna can amplify transmission risk [62].

Recreational activities such as camping, fishing, and hiking in natural areas further contribute to human exposure to *F. tularensis* vectors, increasing the likelihood of zoonotic transmission. In Scandinavian countries, particularly Sweden and Finland, human outbreaks show marked seasonality and are frequently associated with high hare densities and environmental conditions favorable to mosquito activity, as well as increased human recreational exposure, highlighting complex ecological cycles in which environmental and animal factors directly shape human risk [35].

In other European contexts, transmission has been predominantly linked to environmental exposure. In Spain, outbreaks have been reported more frequently in association with farm work and direct contact with rodents and domestic animals, whereas in France, cases have occurred in individuals exposed to rural areas characterized by stagnant water and wet soils, suggesting a significant role of indirect environmental contamination [39,91,97].

Moreover, illegal wildlife trade, which involves the introduction of non-native species into unnatural environments, represents an additional factor in zoonotic spread, exposing populations to risk even in areas where the disease is not endemic. Similarly, international travel and legal trade, through the movement of people and animals between regions, facilitate pathogen transmission and contribute to the geographic spread of diseases previously confined to specific regions, increasing the risk of global outbreaks [39]. A paradigmatic example of the possible consequences of animal movement between regions is the outbreak of tularemia in the black-tailed prairie dogs trapped and sold as pets in an exotic animal facility in Texas [4]. Out of 163 animals diagnosed with tularemia, 47 died and *F. tularensis* was isolated from 23 of the remaining animals [98]. However, animals had already been shipped to US pet shops and abroad, in Europe and Asia, when some of them were found to be infected; a careful analysis established that a human infection resulting from exposure to infected animals was observed in a young animal handler in Texas [99], thus documenting the first case of prairie dog-to-human tularemia transmission.

A key factor linking environmental and human health is climate change, which disrupts ecological balances and alters vector distribution, promoting *F. tularensis* transmission. Variations in temperature and precipitation influence vector habitats, particularly for mosquitoes, increasing the risk of human exposure. Global warming, for example, is associated with rising average annual temperatures and altered seasonal disease patterns [39]. A Swedish study used climate-scenario projections for 2010–2100 and modeled an overall warming of about +2 °C by the end of the century (relative to baseline conditions), predicting a longer tularemia season and a higher future burden in endemic hotspot regions [100]. In addition, extreme climatic events such as floods, droughts, and seasonal shifts may facilitate the spread of zoonotic diseases into new areas, further disrupting natural transmission cycles.

European surveillance data indicate a recent increase in the number of hares undergoing diagnostic testing, with positivity rates ranging from 17.9% to 36.5%, along with occasional reports of infected dogs, supporting the potential role of both wild and domestic species as environmental sentinels for *F. tularensis* exposure [41,101]. However, because animal surveillance is often based on passive monitoring, the true extent of infection is likely underestimated [37].

These data highlight tularemia as a complex ecological phenomenon in which human behavior, wildlife, vectors, and environmental factors interact to determine outbreak emergence. Active enzootic surveillance provides concrete examples of a One Health approach. During the 1999–2000 tularemia outbreak in Kosovo, 64 rodents from five species were tested by enzyme-linked immunosorbent assay (ELISA) for *F. tularensis* lipopolysaccharide (LPS), with positivity detected in the livers of two striped field mice and in 5 of 48 fecal samples. Environmental epidemiological analysis identified rodent feces in food preparation areas (OR 5.8) and high rodent presence around households (OR 5.7) as significant risk factors [102]. Similarly, on Martha’s Vineyard, Massachusetts, where endemic tularemia and recurrent pulmonary outbreaks associated with landscaping and lawn mowing activities have been reported, serological testing of wild mammals showed positivity in 52% of raccoons and 49% of skunks, confirming their role as environmental sentinels, with the sentinel species varying by region [102,103].

The European One Health reports highlight that integrated surveillance (humans, animals, and the environment) allows for earlier identification of outbreaks and the implementation of targeted prevention measures, such as vector control and risk reduction in areas of greatest exposure [37].

The complexity of tularemia necessitates a One Health approach, including structured collaboration and mutual data exchange among public health, veterinary science, ecology, and environmental/climate analysis. Only integrated human–animal–environment surveillance allows for early detection of risk signals and rapid, targeted responses. In this context, wildlife monitoring and ecosystem protection, combined with the reduction in high-impact anthropogenic pressures, help prevent new outbreaks and strengthen the resilience of communities and the environment.

## 7. Ecological Modeling Framework for Tularemia “Wet” and “Dry” Cycles

The different eco-epidemiological cycles of tularemia—the summer “wet” cycle, typically vector-associated, and the terrestrial “dry” cycle, more closely linked to rural environments and animal reservoirs—can be conceptualized as useful heuristic archetypes that may overlap in space and time and therefore require distinct modeling approaches. In “wet” settings (e.g., summer outbreaks in Scandinavia), count regression models (e.g., negative binomial regression) are applied using meteorological and ecological predictors, including proxies for mosquito vector abundance. These eco-climatic models have been shown to explain a substantial proportion of interannual variability in case numbers in high-risk areas and to effectively distinguish epidemic from non-epidemic years [104]. In Sweden, for example, analysis of data from 1984 to 2012 identified spatial clusters of high incidence (using SaTScan [105]) concentrated in a limited number of endemic regions, with cases occurring almost exclusively in summer (July–September). Predictive models based on these factors (temperature, humidity, precipitation, presence of surface water, etc.) are consistent with the “wet” cycle, as they quantify environmental conditions that favor transmission by hematophagous vectors.

Conversely, the “dry” cycle is dominated by terrestrial determinants and the human-rural environment interface. In non-mosquito-dominated settings, infection is primarily associated with direct or indirect contact with wild animals (rodents, leporids) and environmental contamination (e.g., food or water contaminated with excreta). In these more complex scenarios, Bayesian spatiotemporal models are commonly adopted within a One Health framework, enabling the integration of data on animal hosts, environmental factors, and human cases within a single inferential framework. For example, recent studies in Mediterranean settings have correlated vole population outbreaks with subsequent human epidemics, using hierarchical models to describe transmission from animals to humans, supporting the focal and intermittently amplified nature of these transmission regimes [106].

Species Distribution Modeling (SDM) or Ecological Niche Modeling (ENM) approaches are also employed to map the environmental suitability of *F. tularensis*, or of its vectors or hosts, based on climatic, geomorphological, and land-use variables, thereby identifying areas compatible with pathogen persistence. In parallel, integrative tools such as SaTScan for spatiotemporal cluster detection, remote sensing-derived environmental covariates, and genomic analyses (whole-genome sequencing and phylodynamics) further enhance epidemiological investigations. Phylogenomic analyses, for instance, can determine whether strains isolated from distinct outbreaks belong to the same endemic clade. A representative example is Spain, where molecular analyses demonstrated that strains isolated during the 2007 outbreak shared the same genotype as those from 1997, supporting the continuity of an outbreak-associated genotype across events [107]. Collectively, these approaches complement traditional epidemiological models by enabling hypothesis testing on the prevailing transmission cycle, linking outbreak isolates through genomic relatedness when interpreted alongside epidemiological and entomological data, or identifying spatiotemporal clusters consistent with a common environmental source.

### Ecoepidemiological Comparison: Scandinavia, the Balkans and Spain

Three well-documented European regions illustrate the eco-epidemiological variability of tularemia: Scandinavia (Sweden), the Balkans (Kosovo), and Spain (Castile and León). Each region exemplifies a predominant eco-epidemiological profile reported in the literature, with characteristic seasonality and exposure pathways shaped by local environmental conditions.

Scandinavia (Central Sweden): The 2019 outbreak in Gävleborg County (Ljusdal area) is emblematic, as tularemia occurs predominantly in summer and mainly presents in the ulceroglandular form. This context is compatible with a “wet”, mosquito-associated transmission profile, in which mosquito bites represent a major reported exposure route. The local ecosystem, rich in wetlands and forests, provides environmental conditions that favor high seasonal mosquito abundance and human-vector contact [108]. Consequently, warm and humid conditions and the presence of stagnant water are critical drivers, as reflected in predictive eco-climatic models developed in Sweden. In these northern areas, seasonal warning systems based on regression models combining temperature, rainfall, and mosquito abundance indices have been developed and evaluated to anticipate epidemic risk [104]. The typical epidemic pattern is intermittent: years with particularly hot and humid summers show surges in cases, whereas in years unfavorable to vectors, incidence remains low.

Balkans (Western Kosovo): The outbreak in Kosovo, which occurred between 1999 and 2000 in an area not previously regarded as endemic, represents a typical “dry” scenario with predominantly environmental transmission. The 327 confirmed cases, largely oropharyngeal illnesses with fever and cervical lymphadenopathy, were concentrated in rural communities in the western part of the country. Epidemiological investigations indicated that the most likely route of transmission was the consumption of food and water contaminated by rodent excreta, in the context of severe conflict-related sanitation problems. This outbreak, initially suspected to be a possible deliberate event, was later assessed as unlikely to reflect intentional release and was interpreted as a natural outbreak occurring in the setting of post-conflict disruption with compromised sanitation and infrastructure [102,109]. The lack of evidence supporting mosquito-borne transmission as a predominant pathway, together with atypical seasonality (cases occurring even in cold months), is consistent with the “dry” nature of the cycle, in which persistent environmental contamination and the human–rodent interface are key factors. In such contexts, the most suitable analytical approaches are spatiotemporal models combining environmental data (e.g., rodent density, land use) with human case data, often within a hierarchical Bayesian framework to account for underreporting and spatial correlation. The One Health approach is essential, as the joint analysis of veterinary, climatic, and human data enables the identification of risk zones (e.g., through dry-cycle environmental suitability maps) and clarification of transmission mechanisms (e.g., by correlating the distribution of *F. tularensis* in small mammals with human epidemic clusters).

Spain (Castile and León): Tularemia has reemerged in a large agricultural region of Northwestern Spain. Two large, multi-district epidemics (1997–1998 and 2007–2008) involved high numbers of confirmed human cases, marking the recognition of disease activity in a region previously not considered endemic. The predominant cycle is a “dry” zoonotic one: the Spanish epidemics are closely linked to explosive proliferations of the field vole (*Microtus arvalis*), a rodent pest of agricultural fields. Field vole irruptions have been identified as key ecological amplifiers during these events. During outbreaks, human exposure occurred through multiple routes, including inhalation of potentially contaminated dust (during threshing or other agricultural activities), direct contact with infected animals (hares and wild rabbits, whether hunted or handled), and ingestion of contaminated plants or water. Clinical manifestations varied (ulceroglandular, typhoidal, and pneumonic) depending on the route of exposure. Seasonality was predominantly from late summer to autumn, coinciding with rodent population peaks and agricultural harvesting. In Castile and León, integrated modeling approaches were applied: time-series analyses identified temporal patterns in which human epidemic resurgence followed vole population peaks by several months [110], and landscape epidemiology studies demonstrated associations between incidence and agro-ecological and landscape features in areas affected by vole irruptions [111]. Environmental risk maps based on SDM/ENM approaches, incorporating climatic, soil, and land-cover data, were also used to identify areas of greatest suitability for tularemia. In addition, genomic investigations proved informative: molecular analyses of isolates from 1998 and 2008 showed that the strains belonged to the B.Br.FTNF002-00 subtype, widespread in Western Europe, supporting continuity of an outbreak-associated genotype across events and suggesting local persistence rather than repeated reintroductions [107].

**Figure 1:**
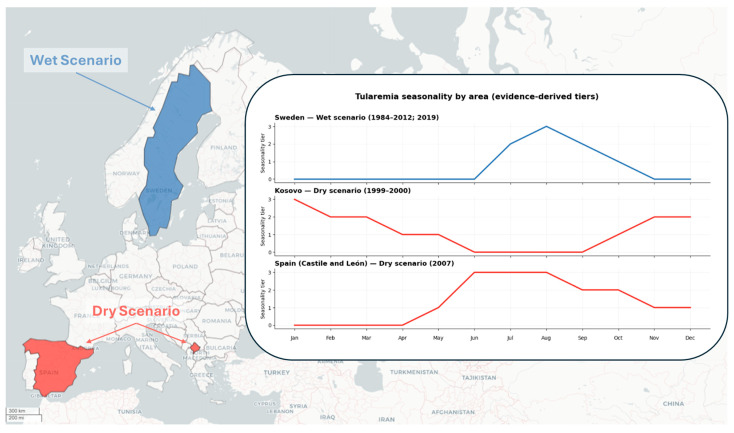
Evidence-based seasonality tiers for tularemia in three European settings representing “wet” and “dry” scenarios. Monthly seasonality is expressed as an ordinal tier from 0 to 3, summarizing both the temporal window and the relative intensity of reported cases. Sweden, considered a wet scenario and based on national data from 1984 to 2012 with the additional 2019 National report, exhibits a predominantly summer pattern, spanning July to September, with a clear peak in August and occasional extension into October [90,94,104,108]. Kosovo, reflecting a dry scenario during the 1999 to 2000 outbreak, shows an occurrence window from October to May, with a peak in January [88,95,102,109]. Spain, specifically Castile and León in 2007, also classified as a dry scenario, presents cases from May to December. The core transmission period extends from June to October, with peak activity concentrated between June and August [93,96,97,107,110,111]. In the figure, lines are color-coded by scenario, with blue indicating wet conditions and red indicating dry conditions.

Figure 1 summarizes the key characteristics of each context, including the predominant transmission cycle, seasonality, exposure routes, salient environmental factors, and the principal modeling approaches applied. Overall, this comparison highlights the marked ecological plasticity of tularemia: depending on local conditions, the disease can assume distinct eco-epidemiological configurations, requiring context-specific surveillance strategies and tailored modeling tools for outbreak prediction and control.

## 8. *Francisella tularensis* Entry the Phagocytic Cells and Virulence Factors

*F. tularensis*, once it has entered the host, is taken up by phagocytic cells, depending on the route of entry. Alveolar macrophages are primarily involved following inhalational exposure, whereas macrophages and dendritic cells are involved after entry via an arthropod bite or skin scarification following contact with infected animal tissue. Protective immunity against *F. tularensis* relies predominantly on robust cell-mediated responses, with coordinated activation of CD4^+^ and CD8^+^ T cells being essential for bacterial clearance and long-term protection. Nevertheless, humoral immunity also contributes to host defense. Antibody responses generated by plasma cells can enhance opsonization and facilitate bacterial uptake by phagocytes, although they are generally insufficient on their own to confer full protection. Together, these findings support a model in which optimal immunity to *F. tularensis* requires the integrated action of cellular and humoral immune mechanisms [112]. The *Francisella*-phagocyte interaction is mediated by a series of receptors, including complement receptors (CR1, CR3, CR4), Fcγ receptors, the mannose receptor, and scavenger class A receptors (SCARA) [23]. CR1 (CD35) and CR3 (CD11b/CD18) have been shown to be involved in the uptake of opsonized *F. tularensis* by human neutrophils following classical complement pathway activation mediated by natural multispecific IgM in non-immune serum [112]. Scavenger class A receptors have recently been demonstrated to participate in the uptake of serum-opsonized *F. tularensis*, thereby facilitating its engulfment [113]. A complementary role is also exerted by the mannose receptor, promoting phagocytic uptake of *F. tularensis*, while, in the extracellular compartment, the lectin pathway of complement is activated through mannose-binding lectin (MBL)-associated serine proteases (MASP 1 and 2) [114].

Following phagocytosis, *F. tularensis* is enclosed within a phagosome, termed the *Francisella*-containing phagosome (FCP) [23]. The residence of *F. tularensis* within the FCP is generally short, particularly when the bacterium is non-opsonized, as it rapidly escapes into the cytoplasm, where it multiplies without early intervention by the immune system. This “early blindness” of the immune system during intracellular bacterial growth within phagocytic cells closely resembles the behavior of *Yersinia pestis* [115]. Escape from the phagosome into the cytoplasm is controlled by several genes clustered within a genomic island known as the *Francisella* pathogenicity island (FPI) [23]. The FPI and the FPI-dependent type VI secretion system (T6SS), together with their effectors, represent the main virulence factors. A four-gene locus within the FPI, termed *igl* (intracellular growth locus) A-D, has been associated with bacterial escape from the phagosome, particularly the protein IglC, which is one of the most highly induced proteins during intracellular infection [23].

The T6SS was first identified in *F. tularensis* in 2009 [116] and is considered a major virulence factor. It functions as an injectisome, an apparatus designed to inject effector molecules that facilitate bacterial escape from the phagosome into the cytoplasm and promote intracellular multiplication, although some recent studies suggest a reduced role of the T6SS in the latter process [23]. The T6SS belongs to the family of contractile injection systems and consists of three main components: a membrane complex anchoring the system to the bacterial cell envelope, a baseplate containing a wedge and spike that organize its structure, and a contractile sheath enclosing an inner tube [23]. Infection with an attenuated strain of *F. tularensis* subsp. *holarctica* (type B) in the mouse liver leads to rapid granuloma formation, contributing to containment of the infection [23].

The LPS of *F. tularensis*, similarly to that of *Y. pestis*, contains a lipid A moiety that is tetra-acylated and composed of longer fatty acid chains (16–18 carbons) than the prototypical hexa-acylated LPS of *Enterobacteriaceae*, which contains chains of 12–14 carbons. In addition, *F. tularensis* LPS is hypophosphorylated. These features result in very poor recognition by the Toll-like receptor 4 (TLR4), a key receptor for macrophage activation, and this lack of activation allows *Francisella* to multiply with minimal engagement of the host innate immune response. This modified LPS therefore represents an additional major virulence factor by effectively impairing macrophage activation [23]. Recognition of *F. tularensis* instead occurs mainly through TLR2 activation, which detects surface lipoproteins; however, *F. tularensis* can downregulate surface lipoprotein expression, thereby further reducing immune recognition and activation through this pathway as well [23].

Another virulence factor is the polysaccharide capsule, whose precise nature, whether identical to the LPS O-antigen or structurally distinct, remains unresolved [23]. In addition, certain membrane phospholipids have been implicated as virulence factors due to their anti-inflammatory properties, as well as secretion systems such as the type I (T1SS) and type II (T2SS) secretion systems [23].

## 9. Host Innate and Adaptive Immune Response

As outlined above, *F. tularensis* initially behaves as a stealth pathogen, remaining largely undetected by the immune system. This is mainly because its LPS, capsule, and membrane phospholipids are very poor activators of phagocyte receptors, thereby allowing undisturbed intracellular replication within phagocytic cells. Once in the cytosol, *F. tularensis* is sensed by cytosolic DNA sensors such as cyclic GMP-AMP synthase (cGAS), which activates the cGAS-stimulator of interferon genes (STING)-interferon regulatory factor 3 (IRF3) axis, leading to the production of type I interferons (IFNs). Type I IFNs act predominantly as anti-inflammatory immune modulators by inhibiting the production of interleukin (IL)-12 by human dendritic cells and IL-17 by γδ T cells in murine models of tularemia [23].

However, type I IFNs may also activate the IRF1-guanylate-binding proteins (GBPs)-absent in melanoma 2 (AIM2) axis, ultimately leading to caspase-1 activation and release of IL-1β and IL-18, thus exerting a pro-inflammatory effect [23]. *F. tularensis* exerts immunosuppressive activity not only at the level of human macrophages but also in neutrophils [23].

Despite being an intracellular pathogen, *F. tularensis*-specific circulating antibodies appear to play a protective role in tularemia. Nevertheless, cell-mediated immunity is crucial, requiring the coordinated involvement of both CD4^+^ and CD8^+^ T cells [23]. In analogy with *Y. pestis*, *F. tularensis* behaves as a stealth pathogen during the early phase of infection, whereas in the later stages, the disease may progress toward dysregulated sepsis with high mortality if left untreated.

**Figure 2:**
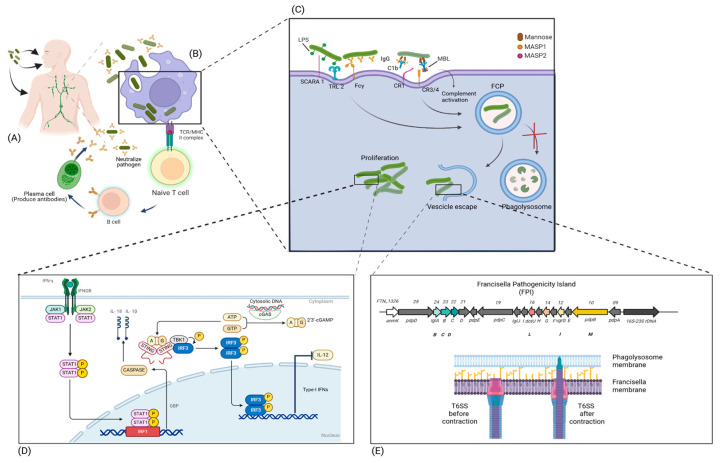
(**A**) *Francisella tularensis* infection: Routes of transmission. (**B**) Macrophage infection and host immune response. (**C**) Intracellular life of *Francisella tularensis* from phagolysosome to cytoplasm. (**D**) Intracellular metabolic pathways for proinflammatory interleukin release. (**E**) *Francisella* pathogenicity island (FPI) and T6SS secretory virulence factor. Created in BioRender. Di Spirito, M. https://BioRender.com/s73tj2e (accessed on 17 February 2026).

Figure 2 depicts *F. tularensis* transmission routes and cellular host interaction.

## 10. Modes of Transmission and Clinical Forms of Tularemia

Tularemia is a zoonosis in which humans are incidentally infected, mainly through the bite of arthropods, particularly ticks and mosquitoes, by direct contact with infected animals or animal materials through the skin, conjunctiva, or oropharyngeal mucosa, by the ingestion of contaminated food or water, or inhalation of contaminated dust or aerosols. *F. tularensis* is highly infectious even at doses as low as 10 or 25 bacteria, via intracutaneous or inhalational routes, respectively [117]. The organism can survive for several weeks in the natural environment [118].

Tularemia presents with several clinical forms, all of which are determined by the route of infection with *F. tularensis*. The most common presentation is the ulceroglandular form, which results from infection following the bite of ticks, mosquitoes, or other arthropods, i.e., transcutaneous transmission, and is characterized by regional lymphadenopathy and an ulcer at the site of entry. In a minority of cases, the ulcer may be absent; these presentations are therefore referred to as glandular forms and are characterized solely by lymphadenopathy. Infection acquired through ingestion of contaminated food or water leads to the oropharyngeal form, whereas direct contact transmission may cause the oculoglandular form, and inhalational exposure results in pneumonic tularemia, which represents the most severe clinical manifestation. Another severe presentation is the typhoidal form, for which the route of infection is often difficult to identify. Possible complications include skin rashes, erythema nodosum, soft tissue abscesses, lymph node suppuration, otitis media, meningitis, and brain abscesses [119].

The ulceroglandular form is the most frequent clinical presentation and may occur with all *Francisella* subspecies. The average incubation period of tularemia is 3–5 days, although durations of up to 2–3 weeks have been reported. Initial symptoms are nonspecific and include fever, chills, fatigue, headache, arthralgias, general malaise, and nausea. In the ulceroglandular form, an ulcer is often present at the site of infection; it may initially appear as a papule, subsequently evolving into an inflammatory pustule with eventual ulceration, or it may heal spontaneously within approximately one week [29]. In this form, timely initiation of appropriate antibiotic therapy may prevent progressive lymph node enlargement and suppuration, a complication that prolongs recovery and occurs in over 20% of cases. Late initiation (>2 weeks) of adequate antibiotic treatment is associated with this complication [11]. In type A tularemia, mortality associated with the ulceroglandular form has been reduced to 1–2%, compared with 5–10% in the pre-antibiotic era, whereas mortality in type B tularemia is virtually absent [94].

The oculoglandular form results from infection acquired through direct contact and presents as a severe, painful unilateral conjunctivitis accompanied by enlargement of the preauricular, submandibular, or cervical lymph nodes, which may occasionally be so marked as to alter facial contours. The oropharyngeal form, caused by ingestion of food or water contaminated with infected animal excreta or carcasses, is characterized by stomatitis, pharyngitis of the mucosa, and possible intraoral ulceration with cervical lymphadenopathy. Rarely, intestinal involvement may occur. In both presentations, symptoms are generally accompanied by abrupt onset of fever, chills, malaise, arthralgias, and myalgias [118].

Pneumonic tularemia results from inhalation exposure and may present with or without pneumonia, in approximately equal proportions. In type A pneumonic tularemia, onset is typically sudden, with chills, fever, dyspnea, cough, chest pain, and profuse sweating in cases of pneumonia, or high fever and marked systemic compromise in typhoidal presentations without pneumonia. Mortality in the pre-antibiotic era was high, ranging from 30% to 60%. Tularemia is a debilitating disease with a prolonged clinical course lasting weeks to months, particularly in the presence of complications. A worse prognosis is observed in immunocompromised patients and in older individuals. Patients with tularemia do not require isolation and should be managed using standard precautions, as no clear evidence of human-to-human transmission has been demonstrated [118].

## 11. *Francisella tularensis* as a Possible Biological Weapon

The characteristics of high transmissibility at low bacterial doses, particularly via the inhalational route, ease of dissemination, environmental persistence for weeks, severity of the clinical disease up to death, frequent diagnostic delay, and the lack of an approved safe and effective preventive vaccine have long supported the view that *F. tularensis* may represent an ideal biological weapon, especially if antibiotic-resistant strains were to be selected for use. For these reasons, it has been included among the most dangerous Category A bioterrorism agents by the U.S. Centers for Disease Control and Prevention (CDC) [120].

Between 1932 and 1945, at Unit 731 in Manchuria, Japanese General Shirō Ishii tested several microorganisms as potential biological weapons, including *F. tularensis*. After World War II, both the United States and the former Soviet Union conducted extensive offensive biological weapons programs, including work on tularemia, involving the development of antibiotic- and vaccine-resistant strains [121]. The United States unilaterally terminated its offensive biological weapons programs in 1969 following a presidential order issued by President Richard Nixon, whereas the former Soviet Union continued these activities until the 1990s, despite being a co-depositary state of the Biological Weapons Convention launched in 1972 [121].

A World Health Organization (WHO) publication in 1970, *Health Aspects of Chemical and Biological Weapons*, modeled the impact of the release of 50 kg of dried *F. tularensis* particles over a city of five million inhabitants, assuming the use of an antibiotic-sensitive strain. The model estimated approximately 250,000 incapacitated individuals and 19,000 deaths [122]. According to a 1997 CDC estimate, deliberate aerosol exposure of 100,000 people to *F. tularensis* would result in an estimated societal cost of approximately USD 5.4 billion [123].

However, the epidemiological characteristics of deliberately released infections differ substantially from those of naturally occurring disease. Deliberate dissemination typically produces outbreaks in unusual geographic locations or seasons, with rapid, temporally concentrated spread, high attack rates, and often worse prognosis, particularly if antibiotic-resistant strains are involved [118]. Based on careful analysis of these and other parameters, the tularemia outbreak in Kosovo between 1999 and 2000, which was initially suspected to be the result of deliberate wartime release of *F. tularensis*, was ultimately considered unlikely to have been intentional, given the low scores obtained across these assessment criteria [109].

That outbreak resulted in 327 serologically confirmed cases, all clinically characterized by pharyngitis and cervical lymphadenopathy, most likely due to ingestion of food or water contaminated by rodents. No deaths were reported [102]. Nevertheless, during the first decade of the 21st century, Kosovo recorded the highest tularemia incidence in Europe, at 5.2 cases per 100,000 inhabitants [124], confirming that war-related socioeconomic disruption strongly influenced tularemia epidemiology, even creating favorable conditions for disease emergence in geographic areas where it had not previously been reported [12]. A similar pattern was observed in Bulgaria between 1997 and 2005, when 285 tularemia cases were reported, 275 (96.5%) of which presented as oropharyngeal forms, most likely associated with food or water contamination [125]. This form of tularemia has also re-emerged in nearby Turkey during roughly the same period [124].

## 12. Diagnosis

The diagnosis of tularemia is challenging, as its clinical presentation is largely nonspecific, the disease is relatively rare even in endemic areas, and cases usually occur sporadically rather than as outbreaks, except during rare natural events or following deliberate release as a biological weapon. Although culture is considered the gold standard for diagnosis, *F. tularensis* is a fastidious organism and extremely infectious even at very low infectious doses. Moreover, cultivation of *F. tularensis* poses substantial biosafety concerns due to its high infectivity at low *inoculum*. For these reasons, handling and culture should be performed in biosafety level 3 (BSL-3) facilities, particularly when infection with the subspecies *tularensis* is suspected [117]. These limitations mean that culture is used in no more than 10% of cases [26].

According to the WHO, tularemia cases are classified as *suspect*, *presumptive*, or *confirmed* (Table 1). A *suspect* case is defined by a compatible exposure history together with clinical symptoms consistent with tularemia. A *presumptive* case includes suggestive clinical symptoms associated with the detection of *F. tularensis* antigen or DNA in a biological sample, or with a single positive serological result. A *confirmed* case requires either identification of *F. tularensis* in culture or antigen or DNA detection, or serological confirmation based on paired serum specimens showing a fourfold difference in antibody titers using tube or microagglutination assays, or a statistically significant increase in titers when ELISA is used, with at least one serum sample testing positive [117].

Confirmatory laboratory diagnosis relies on a combination of culture-based methods, which remain the gold standard, direct detection of bacterial antigens or nucleic acids, and serological testing. Culture-based procedures are hazardous because of the high infectivity of *F. tularensis*, whereas antigen or DNA detection and serology are safer alternatives (Table 1) [117].

Serology is widely used. Specific antibodies generally become detectable between 10 and 20 days post-infection. Standard serological tests include tube agglutination and microagglutination assays [126], which primarily detect IgM antibodies directed against several bacterial antigens, including FopA, LPS, outer membrane carbohydrate-protein fractions, and whole killed bacterial cells. ELISA is also widely used, offering high sensitivity and the ability to detect not only IgM but also IgG and IgA antibodies [127,128]. It should be emphasized that the presence of IgM should not be interpreted as a marker of recent infection, as IgM antibodies may persist for prolonged periods [117]. A significant increase in antibody titers is defined as at least a fourfold rise between two samples collected 1–2 weeks apart for tube or microagglutination tests, whereas for ELISA a significant increase corresponds to a rise greater than two to three standard deviations.

Given the nonspecific early symptoms of potential bacterial agents transmitted by inhalation, such as anthrax, plague, and tularemia, and the need for timely diagnosis, a Luminex assay has been developed for simultaneous immunodetection of these three Category A biological agents [129].

Culture remains the gold standard for diagnosis and represents a valuable resource for molecular epidemiology and subtyping. *F. tularensis* requires cysteine- or cystine-supplemented media for growth. Among these, cystine heart agar supplemented with 9% chocolatized blood (CHAB) is characteristic, yielding green, opalescent, raised, shiny colonies after 24–48 h of incubation, which allow presumptive identification of *F. tularensis*. Mouse inoculation may also be used for isolate recovery. Samples from ulcers frequently yield pure cultures, whereas other clinical specimens may contain contaminating bacteria; in such cases, CHAB supplemented with antibiotics (CHAB-A) can significantly improve recovery rates [130].

Antigen detection may assist in identifying *F. tularensis* in clinical specimens or in isolates recovered in culture through direct fluorescent antibody staining using a fluorescein isothiocyanate (FITC)-labeled rabbit antibody directed against whole killed *F. tularensis* cells [117]. This approach may also be performed using slide agglutination or immunohistochemical staining with monoclonal antibodies directed against LPS in formalin-fixed tissues [131].

Several PCR-based methods have been described for molecular detection of *F. tularensis*. PCR is particularly useful when organisms are non-culturable or when culture is not recommended due to biosafety concerns. Most PCR assays target the *FopA* or *tul4* genes encoding outer membrane proteins [132,133]. The *tul4* PCR assay has been validated using ulcer specimens from patients with ulceroglandular tularemia [134]. Multitarget real-time TaqMan PCR assays with improved specificity and speed have also been developed [135], targeting the IS*Ftu2* element, as well as *23 kDa*, *FopA*, and *tul4* genes. These assays have been applied for diagnosis of *F. tularensis* types A and B [136] and, more recently, for *F. tularensis* subsp. *mediasiatica* [137].

When the diagnostic process is not specifically directed toward *F. tularensis*, 16S rDNA sequencing may be useful [138]. Universal 16S rDNA primers can identify isolates obtained from culture, whereas *Francisella*-specific 16S rDNA primers are required for genus-level phylogenetic analysis and strain identification [25].

Matrix-assisted laser desorption/ionization time-of-flight (MALDI-TOF) mass spectrometry (MS) currently shows limited reliability in identifying *F. tularensis* when commercially available reference databases are used, as these often lack representative spectra for this species. Accurate identification generally requires specialized or in-house curated libraries. In addition, manipulation of viable cultures of this highly infectious pathogen entails significant biosafety concerns, further limiting the routine application of MALDI-TOF MS [139,140].

## 13. Prophylaxis and Therapy

### 13.1. Vaccines

Currently, no approved vaccine for tularemia is available in Western countries, nor have immune correlates of protection been clearly defined. This situation is analogous to that observed for other intracellular pathogens, for which the classical correlate of protection represented by pathogen-specific antibodies, as seen in many viral and bacterial diseases [141], is insufficient or ineffective [142]. In intracellular infections, the role of circulating antibodies is generally limited, whereas cell-mediated immunity is pivotal, as demonstrated by the only available vaccines against other intracellular pathogens, such as BCG for tuberculosis [143] and Ty21a for typhoid fever [144].

The search for a tularemia vaccine is supported by the observation that individuals who recover from the disease appear to remain well protected, either achieving full immunity or developing milder disease upon reinfection. Early vaccine development efforts therefore focused on killed vaccines, considered safer than live attenuated vaccines, which carry a theoretical risk of reversion to virulence. In 1942, Foshay et al. developed a formalin-inactivated or heat-killed bacterial vaccine, which proved to be highly reactogenic and poorly protective [145]. In contrast, a phenol-inactivated vaccine was able to induce some protection in macaques [146] (Table 2).

Before World War II, during roughly the same period in which Foshay and colleagues were developing killed vaccines in the United States, a live tularemia vaccine was developed in the former Soviet Union from a type B *F. tularensis* subsp. *holarctica* strain. This vaccine was administered to more than 60 million individuals during World War II and demonstrated partial efficacy with an acceptable level of reactogenicity [147]. In 1956, despite the Cold War and the existence of active offensive biological weapons programs in both countries, including tularemia-related research, two samples of the live attenuated vaccine were transferred from the Russian Institute of Epidemiology and Microbiology (Gamaleya Institute, Moscow, Russia) to the US Army Medical Research Institute of Infectious Diseases (USAMRIID) at Fort Detrick, Frederick, MD, USA.

Passage of this live attenuated vaccine on peptone–cysteine agar yielded two colony phenotypes, distinguished by color (blue and gray), with the blue colonies being more virulent and more protective than the gray ones [148]. This live vaccine was further characterized, and the blue colony variant was designated the live vaccine strain (LVS). LVS was subsequently used as an investigational new drug (IND) to protect laboratory workers at USAMRIID actively involved in research on *F. tularensis*, given the organism’s extremely high infectivity.

A retrospective comparative analysis of tularemia cases among USAMRIID employees during the period 1950–1959, when protection relied on the Foshay killed vaccine, and during 1960–1969, when LVS was used, demonstrated a significant reduction (*p* < 0.001) in incidence of the typhoidal form of tularemia, from 5.7 to 0.27 cases per 1000 employee-years at risk. No significant difference was observed for the ulceroglandular form; however, clinical manifestations were milder in individuals vaccinated with LVS than in those who had received the Foshay killed vaccine [149].

**Table 2:** Vaccine and antibody prophylaxis.

Vaccines	Killed	Live	Subunits
	Formalin-inactivated or heat-killed bacterial vaccine: highly reactogenic poorly protective [145]. Phenol-inactivated vaccine: some protection to macaques [146]. Inactivated LVS with IL-12 as adjuvant by inhalation almost fully protected mice against a fatal inhalation challenge [150]. Mice immunized via inhalation route with paraformaldehyde- inactivated LVS + cholera toxin B: protected through a Th1 type-mediated mechanism [151].	LVS from type B *F. holarctica* strain blue bacterial colony: quite reactogenic, but effective even against typhoidal [149] and partly against pneumonic tularemia [147]. GMP LVS not substantially different from 1962 LVS [152]. Promising are live vaccines obtained by deletion of genes encoding virulence factors, such as *cplB* [153] *guaBA* [154] and *aroD* [155].	Tul4 and FpoA proteins with CpG adjuvant may induce specific cell-mediated and humoral immunity [156]. Polysaccharide antigen O of LPS linked to different protein components (tetanus toxoid, BSA, and *Pseudomonas aeruginosa* toxin A): highly immunogenic, but only partially protective [142].
**Antibodies**	**Polyclonal**	**Monoclonal**	
	Animal immune sera substantially failed to register a clear antibody-induced protection [157,158,159]. Mice immune sera transferred to other mice 24 h before a fatal inhalation challenge were fully protective. The protection was dependent on FcγR, IFN-γ, neutrophils and macrophages, but not complement [160].	Monoclonal antibodies against LPS LSV are fully protective if ip or in administered within one hour of lethal LVS intradermal challenge [161]. A monoclonal antibody anti-LPS LVS fully protects against a challenge with LVS and partly against a challenge with Schu S4 strain [162].	

LVS = Live Vaccine Strain; Tul4 and FpoA = Outer membrane proteins of *F. tularensis*; GMP = good manufacturing practices; FcγR = Receptor for the fragment crystallizable of IgG; IFN = Interferon; LPS = Lipopolysaccharide; CpG = cytosine–phosphoguanine-containing oligonucleotide; ip = intraperitoneally; in = intranasally; BSA = bovine serum albumin.

Moreover, although LVS-induced antibody responses decline over a few years, specific cell-mediated immunity persists for at least three decades, as demonstrated by antigen-stimulated IFN-γ release from CD4^+^ and CD8^+^ central memory T cells in vaccinated individuals, with no significant differences between recently vaccinated subjects and those vaccinated many years earlier [163]. While both killed and live vaccines provide protection against ulceroglandular tularemia, the live vaccine alone is only partly protective against the more severe pulmonary tularemia, as observed in volunteers exposed to as few as 10–50 organisms of the *F. tularensis* type A Schu S4 strain [147]. Despite these encouraging results, LVS has never been approved by the Food and Drug Administration (FDA), due to its limited efficacy against pulmonary tularemia and concerns regarding potential reversion to virulence, given that the mechanisms underlying its attenuation remain largely unknown [164].

The absence of a safe and effective live vaccine against an infectious agent that, although currently not epidemiologically alarming, is potentially fatal and characterized by low-dose high infectivity and environmental persistence, features that make it an attractive biological weapon, represents a major vulnerability. Consequently, research efforts have focused on developing safer and more effective vaccines, largely by improving LVS, which has demonstrated the highest protective efficacy. These approaches include the use of inactivated and adjuvanted formulations or the LVS preparation in accordance with good manufacturing practices (GMP).

Trials using inactivated LVS combined with interleukin-12 (IL-12) as an adjuvant have been conducted. Mice immunized via the inhalational route with this formulation showed 90–100% protection following a lethal inhalation challenge, with protection largely dependent on secretory IgA, as all mice congenitally deficient in secretory IgA succumbed to infection [150]. The importance of adjuvants in shaping the immune response was further demonstrated by studies showing that mice immunized via the inhalational route with paraformaldehyde-inactivated LVS combined with cholera toxin B developed a Th1-mediated protective response that overcame the requirement for secretory IgA, which was otherwise essential when the vaccine was administered without an adjuvant [151].

Efforts have also been made to revitalize LVS in compliance with GMP. A Phase 2 comparative study (NCT01150695) evaluating traditional versus GMP-revitalized LVS showed no significant differences between the two preparations in terms of local reactogenicity (the vaccine is administered by scarification, similarly to smallpox vaccination), systemic reactogenicity, or humoral immunogenicity, which was high for both formulations (approximately 94% by microagglutination testing) [152].

Additional Phase 2, uncontrolled, open-label studies of LVS administered to at-risk laboratory workers under IND application 157 were conducted by USAMRIID. Two independent studies following similar protocols were performed: the first (NCT00584844) enrolled 405 at-risk laboratory workers from biosafety level 3 laboratories between 2004 and 2008, while the second (NCT00787826) included 170 laboratory workers vaccinated and followed up between 2009 and 2017. When analyzed jointly, the results were favorable, showing good overall safety with no severe adverse events and 100% immunogenicity as assessed by “cutaneous take” reactions. In the first study, all participants except three showed positive serology (microagglutination titer > 1:20). In the second study, a ≥4-fold increase in antibody titers was observed in 95% of participants. Despite LVS having been produced in 1962, it retained full immunogenicity, consistent with these findings and with the broader literature. The comparative Phase 2 analysis with GMP-revitalized LVS similarly demonstrated no substantial differences compared with the original LVS formulation [152,165].

One promising strategy for developing a safer and more effective live vaccine involves targeted deletion of genes encoding virulence factors, such as clpB, which encodes a highly conserved AAA+ chaperone stress protein with immunosuppressive properties [153]. Δ*clpB* mutants of both LVS and Schu S4 have been shown to be less reactogenic and more protective, provided that reduced challenge doses of the Schu S4 type A strain are used [166,167,168]. Additional gene deletions explored for attenuation in live vaccine candidates tested in rodents or rabbits under the “Animal Rule” include Δ*guaBA* (encoding enzymes involved in guanosine monophosphate synthesis, whose deletion results in replication defects [154]) and Δ*aroD* (encoding enzymes of the shikimate pathway responsible for aromatic amino acid biosynthesis in bacteria and plants [155]), in addition to Δ*clpB*, all generated in the Schu S4 type A background to assess safety and protective efficacy in animal models translatable to humans [169]. These mutants appear highly promising, particularly Δ*aroD* and Δ*guaBA* in rabbits, which represent a disease model more closely resembling human tularemia than murine models. Moreover, a potential correlate of protection may be identified in antibody responses directed against the LPS polysaccharide O antigen, which shows a strong correlation with protective efficacy [169].

Subunit vaccines based on peptide cocktails from outer membrane proteins, such as Tul4 and FpoA, combined with adjuvants, such as CpG, have been successfully tested experimentally, demonstrating the ability to activate both cell-mediated and humoral immunity [156]. Glycoconjugate vaccines composed of LPS polysaccharide O antigen linked to different protein components, such as tetanus toxoid, bovine serum albumin, and *Pseudomonas aeruginosa* exotoxin A, were highly immunogenic, but only partially protective [142]. Moreover, nanoparticle vaccines have also been explored, based on the positive experience of the mRNA vaccines developed and used during the recent COVID-19 pandemic and the awareness that the possibility of delivering the vaccine into cells may allow recapitulation of natural infection [142]. However, despite the strong efforts in the search for an effective vaccine, complete protection against a pulmonary infection with *F. tularensis* type A still does not appear to be an achievable near-term goal.

### 13.2. Protective Antibodies

Although *F. tularensis* is generally considered an intracellular pathogen, it has recently been observed to have a significant extracellular phase, thus providing a rationale for potential antibody-mediated protection [170,171]. Pioneering studies on passive immunotherapy conducted by Foshay in the 1940s involved the administration of sera from different animals immunized with formalin-killed *F. tularensis* to mice prior to challenge with a virulent strain of *F. tularensis* subsp. *tularensis*. Although prolonged time to death was observed, these experiments failed to demonstrate clear antibody-mediated protection [157,158], and similar results were reported by other authors [159].

A clear protective effect of hyperimmune animal sera was observed only when rats were used as the experimental model species, as rats are more resistant to *F. tularensis* infection than mice [172]. When rats were injected with sera from goats or horses immunized with killed *F. tularensis* Schu strain prior to challenge with the live Schu strain, survival reached 87.5%, compared with only 3.3–6.6% in rats receiving normal serum [173]. Larson confirmed these findings by showing that transfer of hyperimmune sera from vaccinated goats or rabbits to rats, mixed with virulent *F. tularensis* subsp. *tularensis* at the time of challenge, resulted in survival rates of up to 70%. However, delayed administration of hyperimmune serum 24 h after challenge was ineffective, and no protection was observed. Notably, the use of human serum from individuals who had recovered from tularemia resulted in 100% protection in this model [173].

Sera from LVS-immunized mice, when transferred to other mice challenged with a lethal dose of LVS, were fully protective, provided that IFN-γ was present; in this context, the IgG fraction, but not IgM, of hyperimmune serum mediated protection [174]. In 2007, it was first demonstrated that specific polyclonal antibodies could also protect against severe pulmonary tularemia. Sera from mice intranasally immunized with a sublethal dose of LVS conferred complete protection to recipient mice against a lethal LVS challenge when transferred 24 h prior to challenge. This protection was dependent on Fcγ receptors, IFN-γ, neutrophils, and macrophages, but not on complement activation [160] (Table 2). Similarly, sera from mice infected with the Schu S4 strain and subsequently treated with levofloxacin for 13 days starting on day 3 post infection fully protected mice challenged with Schu S4 when administered either 4 h before or 24 h after intranasal challenge [175].

Monoclonal antibodies targeting *F. tularensis* antigens have been less extensively studied; however, their protective potential has been demonstrated. A monoclonal antibody directed against LPS LVS provided complete protection when administered intraperitoneally or intranasally within one hour of a lethal intradermal LVS challenge [161] (Table 2). Another anti-LPS LVS monoclonal antibody conferred full protection against LVS challenge and partial protection against challenge with the Schu S4 strain [162] (Table 2). Despite these encouraging experimental results, no monoclonal antibodies are currently approved for tularemia, and no clinical trials are registered on www.clinicaltrials.gov.

### 13.3. Antibiotics

#### 13.3.1. Post-Exposure Prophylaxis

In the event of accidental exposure, antibiotic prophylaxis should be initiated within 24 h and continued for 14 days, using oral ciprofloxacin (500 mg) or doxycycline (100 mg) twice daily [176] (Table 3).

#### 13.3.2. Therapy

In analogy with plague, the first-choice antibiotic treatment for severe tularemia is the parenteral administration of an aminoglycoside, gentamicin 5 mg/kg/day, divided into two doses administered 12 h apart. If available, streptomycin at a dose of 2 g/day, given intramuscularly in two doses 12 h apart for 10 days or longer, depending on clinical response, represents a valid alternative. In less severe forms and in mass casualty settings, oral ciprofloxacin or doxycycline is preferable. Ciprofloxacin at a dose of 800–1000 mg/day divided into two doses may be administered orally or intravenously for 10–14 days. Doxycycline, which is bacteriostatic, is administered orally at a dose of 100 mg twice daily for at least 15 days [176] (Table 3).

In children, gentamicin may be used at a dose of 5–6 mg/kg/day divided into two or three doses, with monitoring of serum concentrations, whereas streptomycin may be administered at a dose of 15 mg/kg twice daily (up to a maximum of 2 g/day). In milder forms and in areas endemic for tularemia caused by *F. tularensis* subsp. *holarctica*, ciprofloxacin may be used at a dose of 15 mg/kg twice daily (up to a maximum of 1 g/day). All these antibiotics should be administered for at least 10 days. In immunocompromised patients, the same drugs and dosages are recommended, but the duration of treatment should be extended, generally up to at least 14 days. If longer treatment courses are required, it is advisable to limit aminoglycoside use to the initial phase and subsequently switch to ciprofloxacin, in order to reduce the risk of aminoglycoside-related adverse events [176] (Table 3).

#### 13.3.3. Characteristics of Antibiotics

##### Aminoglycosides

The aminoglycosides streptomycin and gentamicin may cause significant adverse effects, mainly manifesting as largely irreversible ototoxicity and nephrotoxicity. Although streptomycin should be considered the first-choice agent [177], its exclusive intramuscular administration and limited market availability make gentamicin a practical alternative due to its greater manageability, it may be administered intravenously or intramuscularly. However, gentamicin should theoretically be excluded because of its limited intracellular penetration in vitro, which could represent a drawback for the treatment of an intracellular pathogen such as *F. tularensis*. Nevertheless, in vivo gentamicin is slowly internalized by cells through pinocytosis, thereby retaining the ability to kill intracellular *F. tularensis* [178]. Both streptomycin and gentamicin have been shown to effectively clear the infection without subsequent relapse in most treated patients and significantly reduce mortality [179].

##### Chloramphenicol

Chloramphenicol is a bacteriostatic antibiotic and is currently rarely used for the treatment of tularemia, except in cases complicated by meningitis, owing to its high penetration into the cerebrospinal fluid, which may be advantageous in this specific context [176].

##### Tetracyclines

Tetracyclines may represent a valid alternative to aminoglycosides in milder clinical forms, in which parenteral therapy may be replaced by oral treatment. Doxycycline, like other tetracyclines, is generally well tolerated; however, tetracyclines should not be used in children younger than 8 years, due to the potential interference with tooth development. Tetracyclines are bacteriostatic and therefore do not directly kill microorganisms, relying instead on the contribution of specific cell-mediated immunity, which typically develops after 12–14 days. Consequently, the duration of treatment should be at least 2 weeks [176].

##### Quinolones

Quinolones, particularly ciprofloxacin and to a lesser extent levofloxacin, are very promising and well-tolerated antibiotics and represent first-line treatment for infections caused by *F. tularensis* subsp. *holarctica* (type B). This is supported by evidence from a Spanish outbreak, in which ciprofloxacin showed superior efficacy and tolerability compared with streptomycin and doxycycline [180].

##### Antibiotic Microbial Resistance

To date, no natural resistance to aminoglycosides, chloramphenicol, tetracyclines, or quinolones has been described in *F. tularensis*. In contrast, resistance to erythromycin is well documented in Europe, but not in the United States; for this reason, erythromycin susceptibility is used primarily for epidemiological typing rather than for therapeutic decision-making. Nonetheless, concern remains regarding the potential deliberate use of antibiotic-resistant *F. tularensis* strains as biological weapons [176].

## 14. Tularemia and Plague: Similar Pathogens, Different Bacteria

Here, a comparative analysis of the etiological agents of tularemia and plague is proposed, using a recent review on plague as a reference [181]. Since the discovery of tularemia, striking similarities between the two diseases have been recognized. George McCoy, while investigating a “plague-like” disease in ground squirrels in California during animal surveillance activities, observed that a microorganism distinct from *Yersinia pestis* could be isolated, which he named *Bacterium tularense*. Initially classified within the genus *Bacterium*, it was subsequently placed in the genus *Pasteurella*, the same genus that included *Y. pestis* (formerly *Pasteurella pestis*), reflecting the presumed close relationship between the two pathogens and the diseases they cause.

Only in the 1960s, after both organisms had been reassigned to the genera *Francisella* and *Yersinia*, respectively, did DNA hybridization studies clearly demonstrate that they were genotypically distinct [182]. More recent genomic analyses have further highlighted this divergence, revealing a marked difference in genome size: approximately 1.8 Mb for *F. tularensis* [183] versus 4.65 Mb for *Y. pestis* [184] (Table 4).

Despite these genetic differences, many biological and epidemiological features are remarkably similar. Both diseases are zoonoses with closely related eco-epidemiological patterns that are best addressed through a One Health approach. Both pathogens infect a wide range of mammals, with rodents and lagomorphs serving as principal reservoirs, and both can persist in environmental foci capable of sustaining their life cycles, including interactions with free-living amoebae in soil. A key distinction is that *Y. pestis* is confined to terrestrial foci, whereas *F. tularensis* can also be maintained in aquatic and watercourse-associated environments.

The geographic distribution of the two diseases partially overlaps, with endemic foci in the United States and Eurasia. However, tularemia is nearly absent in the Southern Hemisphere, and *F. tularensis* displays marked intraspecies heterogeneity, with strains circulating in North America and the Northern Hemisphere differing substantially in virulence and clinical severity, in contrast to the more clonal nature of *Y. pestis*.

Transmission routes are broadly similar for both pathogens, including arthropod vector bites, direct contact with infected animals or tissues, and inhalation; ingestion represents an additional route unique to *F. tularensis*. The incubation period is short for both diseases, but plague is associated with significantly greater clinical severity and lethality. Both bacteria are non-motile, Gram-negative, facultative intracellular pathogens that employ a comparable strategy of early immune evasion, allowing relatively undisturbed intracellular replication within macrophages, albeit mediated by distinct virulence mechanisms.

Finally, neither approved vaccines nor specific polyclonal or monoclonal antibody therapies are currently available for either disease. Therapeutically, both pathogens are susceptible to the same antibiotic classes—aminoglycosides, tetracyclines, and quinolones—with aminoglycosides remaining the treatment of choice in severe clinical forms.

While the close similarity between plague and tularemia has been emphasized since the discovery of tularemia and periodically reaffirmed [185,186], the apparent paradox between the profound genetic dissimilarity of *Francisella* and *Yersinia* and the strong similarity in bacterial behavior and clinical manifestations may be explained as an example of convergent evolution among intracellular microorganisms [187]. This striking similarity may also have contributed to the historical unrecognition of tularemia, which may have been obscured by the more aggressive and lethal plague.

Tularemia was officially identified in 1911; however, in Japan and in the former Soviet Union, diseases with clinical characteristics now recognized as tularemia had already been described since the 19th [186] and 18th centuries [10], respectively. Moreover, between 2004 and 2007, the medical hypothesis that the “Egyptian plague” in 1715 BC [188], the “Hittite plague” in the 14th century BC [189], and the biblical “plague of the Philistines” in the 12th century BC [190] represented outbreaks of tularemia was proposed and considered biologically plausible [191].

If this hypothesis were correct, plague and tularemia would have coexisted as early as the second millennium BC. At that time, distinguishing between these clinically similar syndromes would have been nearly impossible, as the higher virulence and mortality of plague likely overshadowed tularemia, and microbiology had not yet developed, thereby precluding the identification of distinct etiological agents. Confirmation of this hypothesis can only be achieved through paleogenomic studies, as demonstrated by the identification of *Yersinia pestis* DNA in remains from the late Neolithic and Bronze Age [192]. Should future paleogenomic analyses confirm the presence of *F. tularensis*, it would suggest that the close similarity between plague and tularemia allowed the less virulent tularemia to remain concealed by plague and effectively unrecognized by humankind for more than three millennia.

## 15. Current Challenges

**Figure 3:**
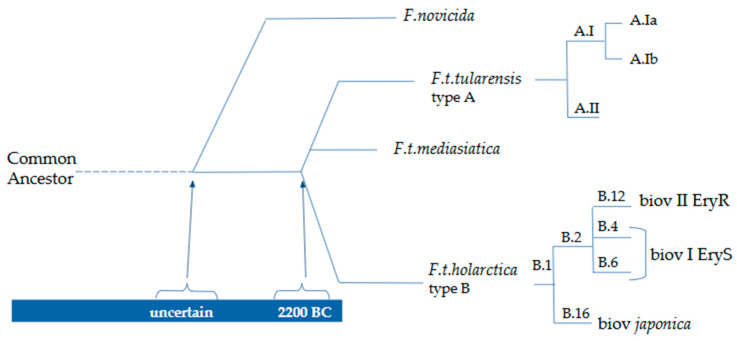
Schematic phylogeny of *Francisella tularensis (F.t.)* from a hypothetical common ancestor.

Many unsolved issues regarding tularemia persist, despite its identification over a century ago, at a time when microbiology had already emerged and was characterized by continuously advancing scientific and technical development that theoretically allowed researchers to face and solve complex problems. However, many questions remain unanswered, beginning with the very origin of *F. tularensis*, which shares some biological characteristics with other intracellular human pathogens, such as *Legionella pneumophila* and *Coxiella burnetii*, even though in other aspects it is relatively distant [4], thus contributing little to clarifying the limited knowledge regarding its origin. In 2005, Svensson et al. identified, through phylogenetic studies, the evolutionary dynamics of the subspecies of *F. tularensis* [193]. Very recently, a new phylogenetic analysis identified China as the geographical origin of *F. tularensis* subspecies *holarctica*, from which it spread worldwide, and estimated that the temporal origin should be placed more than 2 millennia BC, by calculating in that period the presence of the common ancestor of the four clades B4, B6, B12, and B16, thus dating the temporal origin of *F. tularensis* subspecies *holarctica* to more than four millennia ago (Figure 3) [194].

It has been hypothesized, with supporting arguments [29], that it is unlikely that tularemia was a common disease before its discovery because some specific symptoms should not have been overlooked by accurate medical investigations. Nonetheless, this rational argument is only a hypothesis, given that no scientific evidence exists prior to 1911, but only descriptions of suggestive symptomatology. If the Chinese study is also confirmed by paleogenomic analyses, the issue of the origin of the microorganism would be substantially clarified, thus providing support to the hypothesis that the “plagues” described in the second millennium BC could actually have been tularemia.

Moreover, the ecology of the pathogen remains incompletely understood, and many uncertainties persist regarding the possible reservoir(s) and the survival of *F. tularensis* in the active foci out of the epizootic periods.

*F. tularensis* was recognized early after its discovery as highly infective, but interhuman transmission has not been observed for reasons that remain unclear [12], meaning that even patients with pulmonary tularemia do not require isolation.

However, the most relevant current challenge is still the lack of a safe and effective vaccine, probably because of intrinsic biological difficulties posed by intracellular bacteria.

Thus, several pivotal issues remain incompletely understood or unresolved, despite the fact that tularemia has traditionally been considered an ideal category A biological weapon. It is highly advisable that, as occurred after the anthrax letters in the United States in 2001 [170], research programs on tularemia be appropriately stimulated through adequate financial support, so that this potentially fatal infection can be better understood and effectively prevented and managed.

## 16. Conclusions

Plague and tularemia are potentially fatal zoonoses, with plague being the more virulent disease. They are transmitted through similar routes and coexist in many geographic areas, although they sometimes occupy different environmental niches. Tularemia is able to persist in both terrestrial and water-associated foci, whereas plague is restricted to terrestrial ecosystems.

It has recently been hypothesized that tularemia may have been present during the second millennium BC in several Middle Eastern populations, including the Egyptians, Hittites, and Philistines, whose populations were described by historians as being affected by “plagues” but whose clinical descriptions are compatible with tularemia. In particular, for the so-called “Hittite plague”, the well-founded suspicion of deliberate release of infectious material has led to the suggestion that this event may represent one of the earliest documented episodes of biological warfare [190].

The possible confirmation of this hypothesis through paleogenomic studies would raise an intriguing biological question: the long-term coexistence of closely related zoonotic pathogens with markedly different levels of virulence within largely overlapping reservoirs, vectors, and terrestrial geographic foci.

## Figures and Tables

**Table 1:** Case definitions and laboratory diagnosis of tularemia.

Case Definitions	Suspect Case	Presumptive Case	Confirmed Case
	Exposure history with suggestive clinical symptoms	Suggestive clinical symptoms and detection of *F. tularensis* antigen or DNA in a biological sample or with a single positive serological result.	Identification of *F. tularensis* in culture by antigen or DNA detection, or serological confirmation based on paired serum specimens showing a fourfold difference in antibody titers when tube or microagglutination assay are used, or statistically significant increase in titers when ELISA is used, with at least one serum sample testing positive.
**Laboratory diagnosis**	**Culture**	**Molecular**	**Immunological**
	Growth in cysteine/cystine rich agar for 24–48 h: recognition by antigen/DNA identification	Conventional PCR for *FopA*/*tul4* genes Multitarget real-time TaqMan for IS*Ftu2* element, *23kDa*, *FopA*, *tul4* genes	For *Antibody* identification: - Tube or microagglutination (IgM) - ELISA (IgM, IgG, IgA) For *Antigen* identification: - Direct immunofluorescence - Immunohistochemistry

**Table 3:** Post-exposure prophylaxis and therapy of tularemia by antibiotics.

**Post-Exposure Prophylaxis**	Ciprofloxacin 500 mg or Doxycycline 100 mg	orally: twice a day for 15 days starting no more than 24 h after the accident
**Therapy for adults**	Gentamicin 2.5 mg/kg	parenterally: twice a day for 10 days or more according to the clinical response
	Streptomycin 2 g/day	intramuscularly: twice a day for 10 days or more according to the clinical response
	Ciprofloxacin 800–1000 mg/day (in less severe forms and mass casualty settings)	intravenously or orally: twice a day for 10–14 days
	Doxycycline at 100 mg (in less severe forms and mass casualty settings)	orally: twice a day for at least 15 days
**Therapy for** **children**	Gentamicin 5–6 mg/kg	parenterally: twice a day and monitored by serum concentration for at least 10 days
	Streptomycin 15 mg/kg	intramuscularly: twice a day (up to 2 g daily) for at least 10 days
	Ciprofloxacin 15 mg/kg (in milder forms and areas endemic for tularemia type B)	orally: twice daily (no more than 1 g daily) for at least 10 days

**Table 4:** Characteristics of *Francisella tularensis* and *Yersinia pests*.

Characteristics	*Francisella tularensis*	*Yersinia pestis*
Disease	Tularemia	Plague
Transmission	Vector bite, inhalation, contact, ingestion	Vector bite, inhalation, contact
Main Reservoirs	Rodents, Lagomorphs	Rodents, Lagomorphs
Average incubation period	3–5 days	2–3 days
Mortality rate	1–30%	15–70%
Macrophage multiplication site	Cytoplasm	Phagolysosomes
Growth within amoebae	Yes	Yes
Genome size	1.8 Mb	4.65 Mb
Motility	No	No
Intracellular	Facultative	Facultative
Ecosystems	Terrestrial Foci	Terrestrial and water Foci
Approved vaccines	No	No
Approved protective antibodies	No	No
Effective Antibiotics	Aminoglycosides, Tetracyclines, Chloramphenicol, Quinolones	Aminoglycosides, Tetracyclines, Chloramphenicol, Quinolones

## Data Availability

No new data were created or analyzed in this study. Data sharing is not applicable to this article.
